# Comparison of Outcomes in Patients Who Underwent Deep Anterior Lamellar Keratoplasty and Those Converted to Penetrating Keratoplasty

**DOI:** 10.4274/tjo.25675

**Published:** 2017-04-01

**Authors:** Yusuf Koçluk, Emine Alyamaç Sukgen, Ayşe Burcu

**Affiliations:** 1 Adana Numune Training and Research Hospital, Ophthalmology Clinic, Adana, Turkey; 2 Ankara Training and Research Hospital, Ophthalmology Clinic, Ankara, Turkey

**Keywords:** Deep anterior lamellar keratoplasty, penetrating keratoplasty, Descemet’s membrane

## Abstract

**Objectives:**

To compare clinical outcomes of cases who underwent deep anterior lamellar keratoplasty (DALK) and cases who were converted to penetrating keratoplasty (PKP) from DALK surgery.

**Materials and Methods:**

The records of 54 patients for whom DALK surgery was planned and were operated for different diagnoses between March 2013 and June 2015 were retrospectively analyzed. Patients were divided into two groups: group 1 (PKP group) consisted of 23 cases who were converted to PKP due to Descemet’s membrane perforation at any stage of surgery; group 2 (DALK group) consisted of 31 patients whose surgery could be completed as DALK. Preoperative and postoperative follow-up results were evaluated in each group.

**Results:**

Corrected distance visual acuity (CDVA) increased in the postoperative period according to baseline in both groups. However, there was no statistically significant difference in the rates of CDVA increase between the groups (p=0.142). The mean astigmatism measured by corneal topography at final examination was 5.8±2.3 diopters in group 1 and 5.4±1.8 diopters in group 2. The difference between groups was not statistically significant (p=0.430). The groups were not statistically different regarding postoperative pachymetry (p=0.453). The grafts in all 54 patients (100%) were clear at final postoperative examination. There were no statistically significant differences between the groups in terms of postoperative complications.

**Conclusion:**

Similar clinical outcomes were obtained in our study for patients who underwent DALK and those whose procedure was converted from DALK to PKP.

## INTRODUCTION

Penetrating keratoplasty (PKP) has been used as a standard technique in the treatment of corneal stromal pathologies and yields acceptable optical and visual results, as presented in previous studies.^1,2,3^ However, graft failure problems may occur in about 18-34% of cases.^2,3^ Approximately half of graft failures are the result of endothelial rejection.^4^

Deep anterior lamellar keratoplasty (DALK) has been used as an alternative to PKP in the last 15 years in cases with intact endothelium, such as stromal scar, stromal dystrophies, and keratoconus.^5^ This surgical technique conserves the patient’s own endothelial cells. Minimal endothelial cell damage results in longer graft survival postoperatively.^5^ The lower reported intraoperative and postoperative complication rates with lamellar keratoplasty is another reason to prefer DALK.^6^ However, intraoperative complication rates may be slightly higher in the early stages of the DALK procedure. Furthermore, the procedure has a long learning curve, is laborious, and has a longer surgery duration.

The aim of this study was to compare the clinical outcomes in patients who underwent DALK and those whose DALK was converted to PKP intraoperatively.

## MATERIALS AND METHODS

The medical records of 54 patients for whom DALK surgery was planned and who were operated for different diagnoses in our ophthalmology clinic between March 2013 and June 2015 were retrospectively analyzed. The patients were divided into 2 groups. The 23 patients whose DALK was converted to PKP due to Descemet’s membrane (DM) perforation at any stage of the surgery comprised group 1 (PKP group); the 31 patients whose surgery was completed as DALK comprised group 2 (DALK group). The study was approved by the local ethics committee and was conducted in accordance with the principles of the Declaration of Helsinki.

Patients scheduled for DALK whose endothelium was intact and had keratoconus, stroma corneal dystrophy, or stromal scar were included in the study. Patients with additional ocular pathology in the preoperative period and those followed for less than 6 months were excluded. For patients in both groups, preoperative and postoperative 1 day, 1 month, and final follow-up examination data including slit-lamp examination findings (graft transparency, DM attachment, ocular surface and suture problems), intraoperative complications (DM rupture and other problems), and postoperative outcomes (visual acuity, intraocular pressure [IOP], graft status, astigmatism value, corneal thickness values), and postoperative complications (glaucoma, cataract, synechia, rejection reaction, epithelial problems, suture problems, keratitis and interface problems) were obtained from medical records and surgery videos. Corrected distance visual acuity (CDVA) was measured using Snellen chart (in decimal) and IOP was measured using Goldmann applanation tonometry. Astigmatism and corneal thickness values were measured using corneal topography (Pentacam, Oculus, Wetzlar, Germany).

### Surgical Technique

All procedures were performed by the same surgeon (Y.K.) under general or local anesthesia. All patients were initially planned as DALK; cases with intraoperative DM macroperforation as a complication were converted to PKP for the completion of the surgery. The previously described ‘double bubble’ DALK technique was used in cases that underwent complete DALK.^7^

Partial trepanization was performed in 60-80% thickness using a vacuum trepan (Katena Products, Inc., Denville, NJ, USA). A side port was then created in the limbus at 11 o’clock using a micro vitreoretinal (MVR) blade, and a small amount of anterior chamber (AC) fluid was allowed to escape. A few small bubbles were placed in the AC. A 27-gauge needle attached to a 5 ml air-filled syringe was inserted beveled side down through the incision and advanced 3-4 mm toward the center of the corneal stroma. Air was injected onto the DM. When the small bubbles in the AC moved toward the periphery, it was assumed the DM had separated and the big bubble had formed. The anterior stroma was dissected with a crescent knife. The posterior stroma was perforated with an MVR, and the air bubble was emptied. The AC bubble returned to center. Viscoelastic was placed on the DM and the remaining stromal tissue was removed using corneal scissors. In cases where the DM could not be separated with air, we attempted to access the DM using manual dissection. After preparing the graft bed, a donor cornea 0.25 mm larger in diameter than the graft bed was obtained using a vacuum donor punch (Katena Products, Inc. Denville, New Jersey, USA). Removal of the endothelium and DM was facilitated by trypan blue. Grafts with 0.5 mm larger diameter were preferred for patients converted to PKP but without keratoconus. The graft was secured to the graft bed using 10/0 nylon sutures. A 16-point continuous suturation technique was used in all patients. In cases with intraoperative microperforation, the procedure was continued after applying air tamponade to the AC. However, the procedure was converted to PKP in cases with widening perforation and macroperforation. The AC bubbles assisted monitoring of DM integrity throughout the procedure.

### Postoperative Follow-up

Postoperatively, all patients were treated with topical 0.5% moxifloxacin (Vigamox 0.5% sterile ophthalmic solution, Alcon) 6 times daily for 3 weeks and artificial tears for approximately 1 year. In group 1, patients received 0.1% dexamethasone (Maxidex sterile ophthalmic suspension, Alcon) starting at 6 times daily with decreasing doses for 6-8 months; in group 2, patients continued with decreasing doses for 3-4 months. When necessary, elevated IOP was controlled with an appropriate antiglaucomatous agent. When elevated IOP was believed to be associated with steroid use, topical dexamethasone treatment was discontinued for patients in the DALK group, and patients in the PKP group were continued with a lower dose of an agent with lower potency (loteprednol). In patients with slackening of the continuous suture in the early postoperative phase, the suturing was renewed by placing individual stitches. In cases of slackened suture in the late phase, the suture was either removed or, when necessary, removed and replaced with additional interrupted stitches. Complete suture removal was performed after 12-24 months depending on postoperative astigmatism values and whether the surgery was DALK or PKP.

### Statistical Analysis

SPSS for Windows version 16.0 (SPSS Inc. Chicago, USA) was used for statistical analyses. Normality of data distribution was assessed using the Kolmogorov-Smirnov test, and homogeneity was checked using one-way ANOVA. Quantitative variables were presented as mean ± standard deviation, and comparisons between groups were made using a t-test. Pre- to postoperative numerical changes were compared between groups using two-way repeated measures ANOVA. Qualitative variables were expressed as percentages and compared using the chi-square test. P values less than 0.05 were accepted as statistically significant.

## RESULTS

Fifty-four eyes of 54 patients (23 PKP, 31 DALK) were included. The mean age of the patients was 41.1±11.3 years in the PKP group and 38.6±12.8 years in the DALK group. The female:male ratio was 13/10 in the PKP group and 17/14 in the DALK group. There were no significant differences in age or gender distribution between the groups (p=0.457 and p=0.902, respectively). The groups were also statistically equivalent in terms of laterality (p=0.610). Mean follow-up time was 14.0±3.6 (9-20) months in the PKP group and 14.8±5.1 (7-24) months in the DALK group. There was no significant difference between the groups in terms of follow-up time (p=0.492). Preoperative demographic characteristics, indications and postoperative parameters are presented in Table 1.

**Figure 1:**
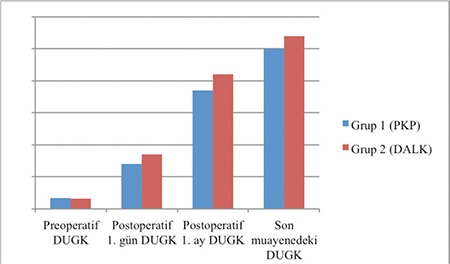
Changes in corrected distance visual acuity in the study groups Preop: Preoperative, Postop: Postoperative, CDVA: Corrected distance visual acuity, PKP: Penetrating keratoplasty, DALK: Deep anterior lamellar keratoplasty

CDVA had significantly improved compared to baseline at postoperative 1 day, 1 month, and at final examination in both groups (p<0.001 for all). However, there was no statistically significant difference in the rates of CDVA increase between the groups (p=0.142, two-way repeated measures ANOVA). Changes in CDVA are presented in Figure 1. IOP values measured preoperatively and postoperatively showed no significant differences in either group, and there was no significant difference between the groups (p=0.456). Mean astigmatism value obtained by corneal topography at final examination was 5.8±2.3 diopters in the PKP group and 5.4±1.8 diopters in the DALK group. There was no significant difference between the groups (p=0.430). Mean central corneal thickness obtained by corneal topography at final examination was 535±35.2 μm in the PKP group and 532±40.1 μm in the DALK group. The difference between the groups was not statistically significant (p=0.453).

**Figure 2:**
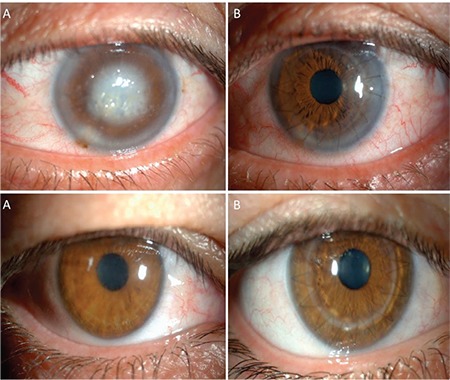
Upper row: Images of a patient with lattice stromal dystrophy who underwent deep anterior lamellar keratoplasty, taken preoperatively (A) and at final postoperative examination (B); bottom row: Images of a patient with lattice stromal dystrophy whose deep anterior lamellar keratoplasty was converted to penetrating keratoplasty, taken preoperatively (A) and at final postoperative examination (B)

Of the patients whose procedure was converted to PKP, DM perforation occurred during trepanization or creation of the big bubble in 6 cases (26.1%), while perforating the posterior stroma in 8 cases (34.8%), and during removal of posterior stromal fragments in 9 cases (39.1%). DM microperforation occurred in 2 patients (6.5%) in the DALK group during suturation, but their procedures were completed as DALK. Double AC was observed on the first postoperative day in 5 patients (16.1%). In these patients, air tamponade was injected to the AC to restore DM attachment and graft transparency was achieved. Clear grafts were observed at final postoperative examination in all 54 patients (100%) in both groups. Pre- and postoperative photographs of one case from each group are shown in Figure 2.

Postoperative steroid-induced glaucoma was observed in 7 patients (30.4%) from the PKP group and 7 patients (22.6%) from the DALK group. The difference was not statistically significant (p=0.515). IOP was controlled in all of these patients with appropriate antiglaucomatous therapy and eliminating or reducing the dosage of topical steroid. None of the patients required glaucoma surgery.

Postoperative cataract developed in 4 patients (17.4%) from the PKP group and 6 (19.4%) from the DALK group. The difference was not statistically significant (p=0.854). Anterior synechia was observed postoperatively in 2 patients (8.7%) in the PKP group and no patients from the DALK group (p=0.094). None of the patients in either group exhibited a rejection reaction postoperatively.

Postoperative recurrent epithelial defect was observed in 1 patient (4.3%) from the PKP group and 3 (9.7%) from the DALK group. Epithelialization was achieved in these cases using conservative methods. All of these patients had a preoperative diagnosis of lattice stromal dystrophy. None of the patients in either group developed keratitis. In addition, a 2-3 mm filamentous foreign body was detected in the paracentral interface of 1 DALK patient (3.2%), but no symptoms or side effects were observed in relation to its presence.

## DISCUSSION

The advantages of DALK include protection of a patient’s own endothelium, less postoperative immune reaction, and shorter follow-up times and steroid use. The closed system of the procedure reduces complications such as intraoperative expulsive hemorrhage, anterior synechia, cataract, and angle narrowing.^6^ With adequate stromal clearing and lowering of the DM, DALK can yield postoperative visual results comparable to those of PKP.^8,9^ In the present study, we also achieved similar postoperative visual acuity results in our DALK and PKP cases. We were able to reach the DM surface in all of our DALK cases with complete removal of the posterior stroma.

In a previous study, mean postoperative astigmatism values after suture adjustment were -2.94 diopters in DALK patients and -3.28 diopters in PKP patients; the difference in astigmatism values was not significant.10 In the same study, postoperative pachymetry values were similar between the groups.10 In our study, astigmatism values were slightly higher postoperatively in both groups. This difference may be explained by the fact that sutures were not adjusted postoperatively in any of our patients. Consistent with the literature, in the present study we observed similar postoperative corneal thickness values between the groups.

Various studies have reported DM perforation during DALK at rates ranging between 4% and 39.2%.^11,12,13^ This rate is associated with surgical experience, keratoplasty indication, and surgical technique.^14^ If DM perforation is not central and is small enough that the integrity of the AC can be maintained (microperforation), the procedure can be completed as DALK, without converting to PKP.^14^ In our study, the DALK procedure was completed in patients with microperforations whose AC integrity was maintained by injecting air. Of the 54 cases in the study, 23 (42.5%) were converted to PKP. In one study, the rate of PKP conversion was 37.9% in the authors’ first 50 cases.^10^

The main advantage of DALK is that it allows the conservation of a patient’s own endothelium. This results in a very low possibility of developing complications such as immunologic reaction or graft failure after DALK.^14^ We observed no graft rejection in any patients in either of our groups. However, one limitation of our study is that the follow-up period was not very long. Another limitation of our study is that endothelial cell count and morphology were not evaluated postoperatively.

In a study evaluating and comparing 10-year outcomes of postoperative complications such as epithelial defect, suture problems, glaucoma, cataract, rejection, and graft separation, the DALK group showed significantly lower rates of postoperative complications.^15^ Minimal endothelial cell damage may allow longer graft survival postoperatively.5 In our study, patients were followed for a shorter time period (maximum 24 months) compared to the aforementioned study, and the postoperative complication rates in the DALK and PKP groups were comparable. However, these similar complication rates could be associated with the fact that the cases included in the present study were the first DALK procedures performed by the authors. Gaining more surgical experience with DALK may result in lower complication rates that are more consistent with the literature. Our results for postoperative graft transparency, visual, and refractive results were comparable to those of the previous study.

## CONCLUSION

In summary, our study yielded similar postoperative clinical outcomes for patients who underwent DALK and those converted from DALK to PKP. Further studies including the evaluation of endothelial cell function and longer follow-up times are needed in order to make a more robust comparison.

## Figures and Tables

**Table 1:**
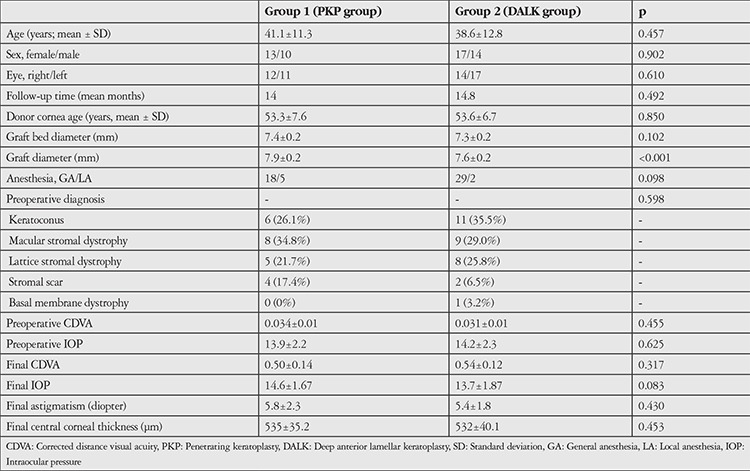
Comparison of demographic, surgical, and pre-/postoperative values for certain parameters between study groups
